# Parameters for estimating the feasibility of implantation of a semi-implantable bone conduction device (SIBCD) in children and adolescents

**DOI:** 10.1007/s00405-022-07752-6

**Published:** 2022-12-01

**Authors:** Jennifer L. Spiegel, Janna de Buhr, Saskia Freytag, Mattis Bertlich, Jan M. Sommerlath Sohns, Martin Canis, Friedrich Ihler, Bernhard G. Weiss

**Affiliations:** 1 Department for Otorhinolaryngology, University Hospital, Ludwig-Maximilians-Universität München, Marchioninistr. 15, 81377 Munich, Germany; 2 German Center for Vertigo and Balance Disorders, University Hospital, Ludwig-Maximilians-Universität München, Marchioninistr. 15, 81377 Munich, Germany; 3 Department of Otorhinolaryngology, Universitätsmedizin Göttingen, Georg-August-Universität Göttingen, Robert-Koch-Str. 40, 37075 Göttingen, Germany; 4 Population Health and Immunity Division, Walter and Eliza Hall Institute, 1G Royal Parade, Parkville, Australia; 5 Department of Medical Biology, University of Melbourne, Parkville, 3052 Australia; 6 Department of Dermatology, University Hospital, Ludwig-Maximilians University of Munich, Munich, Germany; 7 Institute for Diagnostic and Interventional Radiology, Universitätsmedizin Göttingen, Georg-August-Universität Göttingen, Robert-Koch-Str. 40, 37075 Göttingen, Germany; 8 Department of Nuclear Medicine, Medizinische Hochschule Hannover, Carl-Neuberg-Straße 1, 30625 Hannover, Germany

**Keywords:** Semi-implantable bone conduction device, SIBCD, Bonebridge, Radioanatomy, Geometric changes

## Abstract

**Purpose:**

In children and adolescents, preoperative planning for a semi-implantable bone conduction device (SIBCD) is crucial. The geometric changes of the new version of a common SIBCD should enable a higher rate of successful implantation due to its flatter actuator. Thus, this radioanatomic study compared the rate of successful implantation of both device versions at the traditional mastoidal localization and two alternative sites, retrosigmoidal, and parietal, and investigated parameters helping to estimate the feasibility.

**Methods:**

A retrospective analysis of 136 CT scans of 0 to 20-year-old patients, evaluation of demographic parameters, radioanatomy, and assessment of head diameter was conducted. The feasibility was investigated for certain age groups at three implantation sites. Prediction of feasible implantation by means of different parameters was calculated.

**Results:**

A significant higher implantation rate was observed with the new device for all three sites and age groups. The age group of 6–8 years (*n* = 19) had most striking differences with a 58.1% rate of successful implantation with the new device without spacer (80% with spacer) at the mastoidal localization, whereas none with the old implant. Head diameter was identified as the most predictive parameter regarding all implantation sites (mastoidal: *p* = 0.030; retrosigmoidal: *p* = 0.006; parietal: *p* < 0.0001), age for the mastoidal (*p* < 0.0001) and retrosigmoidal (*p* < 0.0001), and gender for the parietal site (*p* = 0.001).

**Conclusion:**

The geometric changes of the actuator lead to a higher rate of successful implantation in all age-groups and all three localizations with reducing the requirement for spacers. Parameters age and head diameter might aid in estimating the rate of successful implantation in young patients and may be a novel tool to assist in the decision-making process for a SIBCD.

**Supplementary Information:**

The online version contains supplementary material available at 10.1007/s00405-022-07752-6.

## Introduction

Hearing rehabilitation with semi-implantable bone conduction devices (SIBCD) is an option in patients with conductive (CHL) or mixed hearing loss (MHL), when hearing aids are not feasible [1]. Utilizing the principle of bone conduction, these devices achieve sound amplification by direct attachment of the actuator to the skull. Via bone conduction, the sound is transmitted by compression and expansion of the skull bone generating shear forces within the cochlea leading to motion of endo- and perilymphatic fluids [2]. The actuator is covered by intact skin and both, information and energy are transmitted transcutaneously via induction from the external audio processor with power supply, from which its description “semi-implantable” derives. One SIBCD, is distributed by MED-EL, Innsbruck, Austria, under the tradename *Bonebridge* and experience with it is well-documented [3]. In countries with CE-certification this SIBCD is approved for patients above 5 years with CHL or MHL and bone conduction thresholds better than 45 dB hearing level [4]; regarding countries with FDA-approval this device can be implanted at an age of 12 years. Traditionally and in case of a normal anatomy, the SIBCD is positioned within the mastoid process by avoiding compression of the sigmoid sinus or dura mater. However, this implantation site may be not suitable for all patients due to anatomy variations or different pneumatization patterns that are, moreover, associated with certain syndromes of various prevalence [5–7]. In cases with altered anatomy, due to a pre-existing tympanomastoid cavity or malformations, two alternative implantations sites are recommended: the retrosigmoidal localization, in which the actuator is placed posteriorly to the sigmoid sinus and caudally to the transverse sinus. Or the parietal localization when the device is implanted dorsocranially to the parietomastoid suture and cranially to the transverse sinus [8–11]. For optimal implantation, which means avoiding the risk of dura mater or sigmoid sinus compression, preoperative planning based on a computed tomography (CT) is recommended [4, 12]. In particular in young patients, preoperative planning is crucial for optimal placement of the actuator and to prevent complications. However, in the very young patients one is hesitant to perform CT scans, since children seem to respond extremely sensitive on radiation [13, 14]. In a study with virtual implantation on CT scans, application was feasible in approximately 80% of the adult cohort, but in the majority of the children under the age of 8 years the drilling depth of the implant bed exceeded the bone thickness. Using the manufacturer’s bone-conduction lifts, an implantation was feasible in 50% of the age group of 6 years and older, and in 100% of the children older than 9 years [15]. Based on the results of this study, the manufacturer applied geometric changes to the actuator and introduced in September 2019 the second generation of the *Bonebridge*, which is flatter but with a wider diameter. To search for further parameters assisting in estimation of the feasibility of implantation in children and adolescents, this radioanatomic study investigated besides the value of age, also the easy to assess parameters head circumference and gender for surgical planning of the implantation of a SIBCD.

## Materials and methods

### Patients, imaging, and ethical standards

In total, 746 consecutive CT scans of the head due to different indications were screened. 136 were included to the study after applying the exclusion criteria: pre-existing implants or other artefacts that compromised the evaluation of the analyzed region, dislocated fracture within the temporal bone or adjacent skull base, abnormal mastoid pneumatization, resolution of more than 0.6 mm slice thickness, and inner or middle ear malformation, since an age-appropriate normal neurocranium was aimed to be assessed in this study (Supplemental Table). Some patients had received multiple CT scans within a short period of time, e.g., for follow-up after surgery or trauma. We included these cases when the CT scans were performed in different age groups, but included a patient only once within the same age group. All scans were screened by a radiologist trained in evaluation of the temporal bone. According to the ethical standards of the Helsinki Declaration the study was approved by the responsible institutional review board [16].

### Implant

**Fig. 1:**
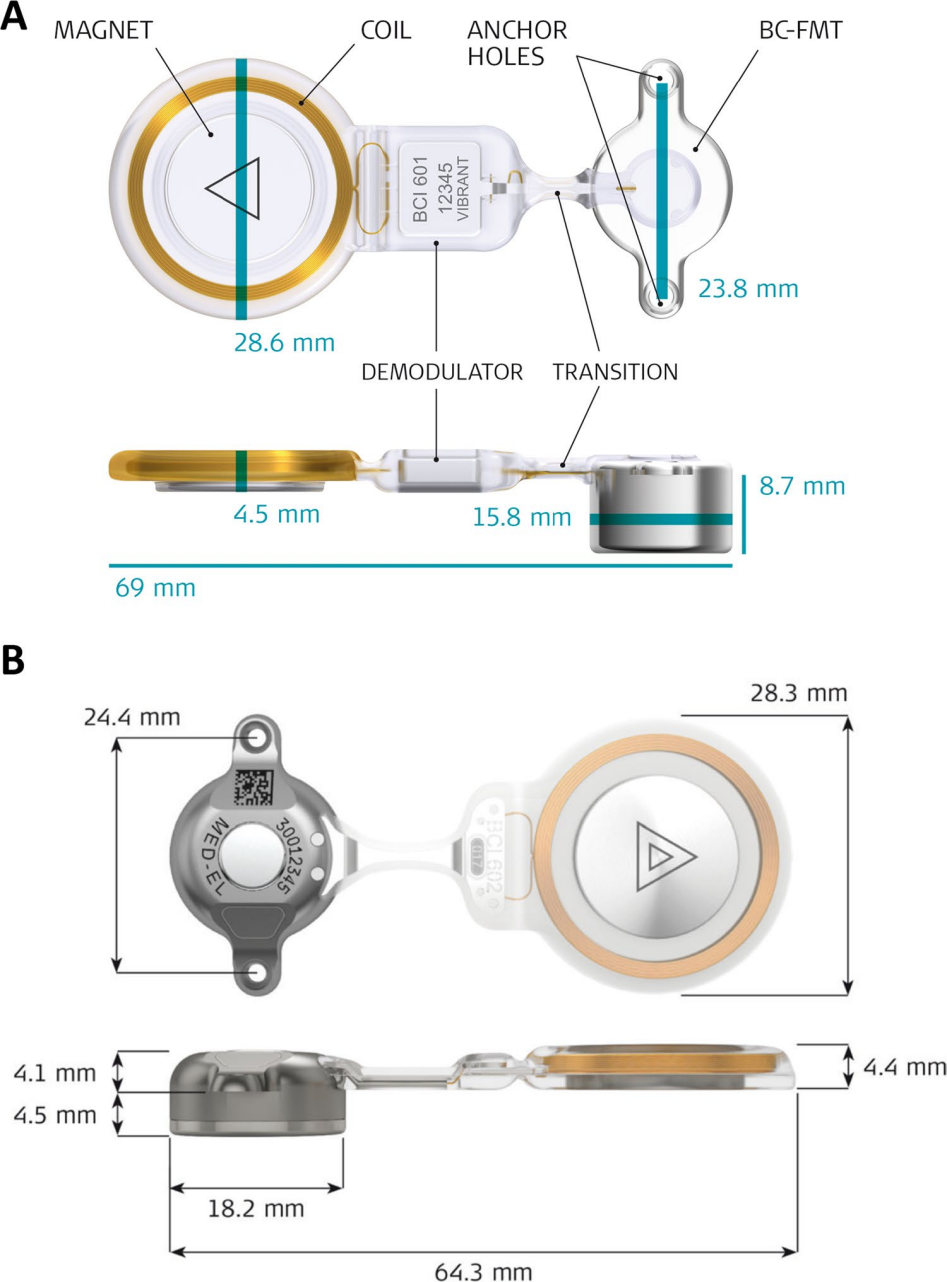
**A** *Bonebridge BCI-601* and **B** the new version: *Bonebridge BCI-602*. The illustrations were provided with kind permission by MED EL GmbH, Innsbruck, Austria. *BC-FMT* bone conduction floating mass transducer

The investigated SIBCD is distributed under the tradename *Bonebridge* by the manufacturer MED-EL (Innsbruck, Austria) and CE-certified since April 4, 2012 (No. I7 12 03 51383 010). In September 2019, the manufacturer introduced the second generation of the device (old BCI-601; new BCI-602; Fig. 1A + B). As depicted in Fig. 1, relevant differences lie in the depth of the actuator which is significantly flatter in the new device. Moreover, the screws delivered with the implant have a different effective penetration depth (BCI-601: 4.5 mm; BCI-602: 3.5 mm). Spacers are available to lift the implant from the skull and reduce implantation depth, which have been changed: with the old device 4 different spacer from 1 mm up to 4 mm, with the new BCI-602 only one type (1 mm) is available.

### Software planning tool and virtual implantation

The software tool *BB Fast View* (CEIT and Tecnun Universidad de Navarra, Donostia-San Sebastian, Spain; http://www1.ceit.es/cg/BBFastView/) was used to analyze the CT scans. After import of the digital template of the SIBCD into DICOM (Digital Imaging and Communications in Medicine) data sets, the assessment was performed in 2D and 3D as described previously [17]. All measurements were performed by the same otorhinolaryngology-trainee, who was supervised by both a senior radiologist and otorhinolaryngologist.

Prior to measuring, randomization for the side to evaluate was done. Qualitative analysis included the feasibility of implantation and considering the need of impression of the dura or sigmoid sinus at a given localization for both implants separately. The thickness of the cortical outer layer for a secure placement of the screws, total bone diameter at the implantation site and thickness of the epicranium, as well as the extent of impression of the dura if necessary, and the distance from the implant to the cochlea were assessed quantitatively. The conduction of these measurements has been detailed previously [17]. In brief, the virtual implant was moved in 3D with axial rotation to achieve optimal fitting of the actuator, as well as its screws, at the traditional mastoidal implantation site and two alternative sites, retrosigmoidal and parietal. A placement without compression of delicate structures, such as the sigmoid sinus or the dura was sought. Since the skin flap superficial to the implant is of a recommended thickness of 7.0 mm, the thickness of the epicranium was assessed at a localization, where the coil was likely to be placed. For optimal fit of the magnet, the manufacturer recommends a thinning of the skin flap when it exceeds the 7.0 mm thickness. Nevertheless, thinning out the flap can be accompanied with postoperative complications, as well.

### Assessment of head circumference

The head circumference was measured at the CT scan with the method of Vorperian et al. which measures the outer diameter of a reconstructed oblique axial section from the glabella to the protuberatia occipitales externa [18]. By applying a non-linear correction factor the accuracy of the estimation by accounting for nonlinear growth rates and sex differences in head growth can be improved.

### Statistical analysis

The statistical approach followed a two-step procedure. First, inferential statistics were used to identify significant covariates followed by the use of predictive statistics: (1) for each implantation site, a logistic regression was fitted to model implantation success by age, head size, gender, and device (consisting of 3 dummy variables for BCI-602 without spacer, BCI-601 with spacer, and BCI-601 without spacer). The logistic regression was fitted using an iterative reweighted least squares approach using the stat package (version 4.0.5) in the statistical software R (version 4.0.5). Covariates were tested for equality to zero using likelihood ratio test and resulting *p* values were corrected using a Benjamini–Hochberg procedure. (2) For each implantation site, utilizing the caret package (version 6.0-88), we then used the significant covariates to generate a prediction model using a fivefold cross validation to estimate accuracy, sensitivity, and specificity. Note that we only generated predictive models for new technology and separately for the use of spacers and no use of spacers.

## Results

### Patient and radioanatomic characteristics

Radiologic data sets of 136 children and adolescents were included into this investigation with 60 female subjects (44.1%). The age at timepoint of radiologic study was 12.5 ± 5.7 years (range 0.67–20.3 years). Head circumference was 53.2 ± 3.1 cm (range 43.8–59.6 cm). The skin thickness of the flap covering the potential implant bed of the coil was similar at all localizations (mastoidal 6.1 ± 1.7 mm; retrosigmoidal 5.8 ± 1.8 mm; parietal: 6.0 ± 2.8 mm). Distance from the implantation site to the cochlear was the shortest for the mastoidal site (36.4 ± 3.0 mm; retrosigmoidal: 49.2 ± 3.3 mm; parietal: 63.9 ± 4.4 mm).

### Feasibility of implantation at the different implantation sites

**Fig. 2:**
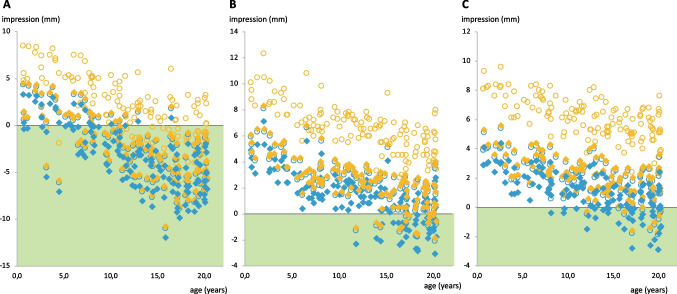
Impression of the dura/sigmoid sinus regarding the three different implantation sites mastoidal, retrosigmoidal, or parietal with or without spacer. **A** Implantation site mastoidal, **B** retrosigmoidal, and **C** parietal. The yellow empty circles depict values for BCI-601 without spacer, blue empty circles BCI-602 without spacer, yellow filled rhombus BCI-601 with spacer (4 mm), and blue filled rhombus BCI-602 with spacer (1 mm). The grey solid line indicates the zero line: all values below this line suggest a feasible implantation at this site

CT scan measurements at the implant localizations mastoidal, retrosigmoidal, and parietal considered a potential affection of the sigmoid sinus and the dura mater comparing the old BCI-601 and new BCI-602. Mastoidal implantation without affecting the sigmoid sinus without spacers was possible in more cases with the new device (BCI-602: *n* = 101, 74.3%; BCI-601: *n* = 45, 33.1%; Fig. 2A). The minimal remaining thickness of the bone covering the sigmoid sinus in this localization was larger on average with the BCI-602 (4.0 ± 3.3 mm; BCI-601: 2.0 ± 1.4 mm). The strength of the cortical outer layer of the bone at the mastoidal site, where screws would grasp securely (Table 1), ranged from 0.7 to 4.7 (mean 1.8 ± 1.1 mm) in all implantable BCI-601 (*n* = 45) and 0.7–8.0 (mean 1.8 ± 0.9 mm) in all implantable BCI-602 cases (*n* = 100).

**Table 1:** Successful implantation BCI-601 (spacer: 4 mm) versus BCI-602: (spacer: 1 mm)

	BCI-601		BCI-602	
	*n*	(%)	*n*	(%)
Without spacer				
Mastoidal	45	(33.1)	101	(74.3)
Retrosigmoidal	0	(0.0)	18	(13.2)
Parietal	0	(0.0)	11	(8.1)
With spacer				
Mastoidal	99	(72.8)	116	(85.3)
Retrosigmoidal	16	(11.8)	32	(23.5)
Parietal	10	(7.4)	37	(27.2)
Secure placement of screws				
Mastoidal	132	(97.1)	133	(97.8)
Retrosigmoidal	41	(30.1)	55	(40.4)
Parietal	57	(41.9)	85	(62.5)

Safe implantation without spacer with the BCI-601 deemed possible only at the mastoidal site. Whereas with the new BCI-602, retrosigmoidal implantation without spacers was feasible in 18 cases (13.2%) and parietal in 11 cases (8.1%; Fig. 2B + C. Regarding feasibility with spacer, the new implant was superior (Table 1 and Fig. 2). Whenever mastoidal implantation was impossible, implantation at the alternative sites was impossible, as well.

### Feasibility of implantation regarding age groups

The age group of infants and toddlers (0–2 years; *n* = 10) showed no feasible implantation of any SIBCD without spacer. However, with spacers, in three individuals (30.0%) mastoidal implantation seemed possible with the BCI-602. The age group of the 6–8 years (*n* = 19) showed no feasible implantation without spacer with the old device, whereas in 8 (58.1%) with the new implant at the mastoidal localization (BCI-602 with spacer 78.9%, *n* = 15). The group of 9–11 years (*n* = 19) showed a mastoidal rate of successful implantation of 78.9% (*n* = 15) without spacer with the new BCI-602, which was reached with the BCI-601 only after application of a 4-mm spacer (78.9%; *n* = 15). In the 12–14 years (*n* = 23) mastoidal implantation deemed possible with the BCI-602 (without spacer) in almost all patients (*n* = 22), whereas with the old device in only 42.1% (*n* = 8). Regarding alternative sites, this was an option in only a few individuals for both implants, respectively. In the oldest and largest age group of the adolescents (15–20 years, *n* = 54) mastoidal implantation was deemed possible without spacer in all patients with the new and after application of 4-mm spacers also with the old device. A feasibility rate of around half of the cohort was reached at the retrosigmoidal (51.9%, *n* = 28) and parietal site (46.3%, *n* = 25) with the new implant with the 1 mm spacer (Table 2).

**Table 2:** Successful implantation regarding the age-groups BCI-601 (spacer: 4 mm) versus BCI-602: (spacer: 1 mm)

	BCI-601		BCI-602		Skin flap	
	*n*	(%)	*n*	(%)	mm	(± SD)
0–2 years, *n* = 10 (7.4%)						
Without spacer: mastoidal	0	(0.0)	0	(0.0)	4.34	(1.16)
Retrosigmoidal	0	(0.0)	0	(0.0)	4.17	(1.41)
Parietal	0	(0.0)	0	(0.0)	4.27	(1.95)
With spacer: mastoidal	0	(0.0)	3	(30.0)		
Retrosigmoidal	0	(0.0)	0	(0.0)		
Parietal	0	(0.0)	0	(0.0)		
3–5 years, *n* = 11 (8.1%)						
Without spacer: mastoidal	2	(18.2)	2	(18.2)	4.25	(0.93)
Retrosigmoidal	0	(0.0)	0	(0.0)	3.60	(0.70)
Parietal	0	(0.0)	0	(0.0)	3.25	(0.58)
With spacer: mastoidal	2	(18.2)	5	(45.5)		
Retrosigmoidal	0	(0.0)	0	(0.0)		
Parietal	0	(0.0)	0	(0.0)		
6–8 years, *n* = 19 (14.0%)						
Without spacer: mastoidal	0	(0.0)	8	(58.1)	4.64	(0.64)
Retrosigmoidal	0	(0.0)	0	(0.0)	3.89	(1.70)
Parietal	0	(0.0)	0	(0.0)	4.12	(0.88)
With spacer: mastoidal	8	(58.1)	15	(78.9)		
Retrosigmoidal	0	(0.0)	0	(0.0)		
Parietal	0	(0.0)	1	(5.2)		
9–11 years, *n* = 19 (14.0%)						
Without spacer: mastoidal	2	(10.5)	15	(78.9)	5.55	(1.22)
Retrosigmoidal	0	(0.0)	1	(5.2)	5.34	(1.18)
Parietal	0	(0.0)	0	(0.0)	5.18	(1.07)
With spacer: mastoidal	15	(78.9)	18	(94.7)		
Retrosigmoidal	1	(5.2)	1	(5.2)		
Parietal	0	(0.0)	3	(15.8)		
12–14 years, *n* = 23 (16.9%)						
Without spacer: mastoidal	8	(42.1)	22	(95.7)	6.88	(1.61)
Retrosigmoidal	0	(0.0)	2	(8.7)	6.57	(1.76)
Parietal	0	(0.0)	1	(4.3)	6.54	(1.44)
With spacer: mastoidal	21	(91.3)	22	(95.7)		
Retrosigmoidal	2	(8.7)	3	(13.0)		
Parietal	1	(4.3)	8	(34.8)		
15–20 years, *n* = 54 (39.7%)						
Without spacer: mastoidal	33	(61.1)	54	(100.0)	7.05	(1.38)
Retrosigmoidal	0	(0.0)	15	(27.8)	7.00	(3.11)
Parietal	0	(0.0)	10	(18.5)	7.58	(1.54)
With spacer: mastoidal	53	(100.0)	54	(100.0)		
Retrosigmoidal	13	(24.1)	28	(51.9)		
Parietal	9	(16.7)	25	(46.3)		

The skin flap over the implant was similar at all three localizations with an increase with advancing age. In 32 of the patients (23.5%) the skin would be needed to be thinned out at the mastoidal localization (retrosigmoidal: *n* = 34, 25.0%; parietal: *n* = 44, 32.4%; Table 2).

Since this SIBCD is approved in patients older than 5 years, we had a closer look at our cohort of younger children: regarding the bone thickness we measured similar values at all sites (mastoidal: 3.0 ± 2.6 mm; retrosigmoidal: 2.7 ± 1.0 mm; parietal 2.7 ± 0.9 mm).

### Correlation of parameter with feasibility of implantation

The logistic regression analysis found the parameters age, gender, and head diameter to be most influential on the success of the implantation depending on the different localizations. The diameter of the head showed significant influence on the successful implantation at all investigated sites. Regarding the mastoidal and the retrosigmoidal site, age, and head diameter were most determining. At the parietal localization, female gender and head diameter had the greatest influence. Investigating a potential influence on the feasibility with regard to the head diameter and new versus old implant, only at the mastoidal localization significant differences between the new and old device regardless with or without spacer were identified. For all other variables and localizations no significant differences were found (Table 3).

**Table 3:** Correlation of parameters with successful implantation

	*p* values
Mastoidal	
Gender	0.388
Age	< 0.0001*
Head diameter	0.030*
Retrosigmoidal	
Gender	0.754
Age	< 0.0001*
Head diameter	0.006*
Parietal	
Gender	0.001*
Age	0.051
Head diameter	< 0.0001*

*Indicate significant values

**Fig. 3:**
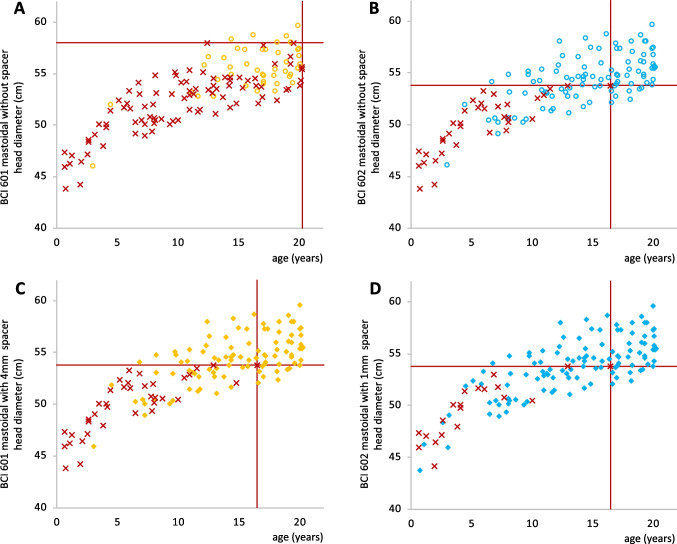
Head diameter versus age regarding mastoidal implantation with or without spacer with regard to the new BCI-602 or old BCI-601. **A** BCI-601 without spacer, **B** BCI-602 without spacer, **C** BCI-601 with 4 mm spacer, and **D** BCI-602 with 1 mm spacer. The yellow empty circles depict values for BCI-601 without spacer, blue empty circles BCI-602 without spacer, yellow filled rhombus BCI-601 with spacer (4 mm), blue filled rhombus BCI-602 with spacer (1 mm), and the red cross non-implantable candidates. The horizontal red solid line indicates the cutoff for the head diameter: all values above this line suggest safe implantation at this site. Cutoff values for head diameter: BCI-601 without spacer: 58.0 cm; BCI-602 with and without spacer, as well as BCI 601 with spacer: 53.8 cm. The vertical red solid line indicates the cutoff for the age: all values beyond this line suggest safe implantation at this site. Cutoff values for age: BCI-601 without spacer: 20.3 years; BCI-602 with and without spacer, as well as BCI-601 with spacer: 16.5 years. For the other localizations (retrosigmoidal and parietal) no cutoff values were observed

Figure 3 depicts the relation of mastoidal feasibility regarding head diameter and age. Without spacer impossible BCI-601 implantation could occur at any age, whereas a safe implantation was found at a cutoff value of 58.0 cm head diameter (Fig. 3A). With spacers, this cutoff value was lowered to 53.8 cm. Regarding age, a safe implantation could be expected at 16.5 years (Fig. 3C). With the BCI-602 cutoff values with/without 1-mm spacer showed no differences (53.8 cm, 16.5 years; Fig. 3B + D). Regarding retrosigmoidal and parietal implantation, no meaningful cutoff values could be identified (Supplemental Figs. 1 + 2).

## Discussion

The present study is the first to evaluate further parameters, besides age, to potentially influence the feasibility of implantation of a SIBCD in children and adolescence in three possible localizations (mastoidal, retrosigmoidal, parietal) and compares these parameters between the old and new version of this specific device. Besides the known correlation of age and successful implantation [15], we found two further parameters (head diameter and gender) which aid in estimating the feasibility in certain age groups depending on the implantation site. Interestingly, head diameter was influential on the successful implantation regardless of the implantation site. Since this parameter is easy to assess, it might be considered to be implemented into the decision-making process for implantation even before routine preoperative planning with the necessity of CT scans. Applying these parameters may aid the decision to wait for further growth before performing a CT scan hastily that would only confirm the implant being not yet suitable. In particular, in the very young patients one is hesitant to perform CT scans of the head, since children seem to respond extremely sensitive on radiation [13, 14]. Therefore, performing an MRI instead and using those parameters to avoid unnecessary radiation exposure could be a potential future approach in the decision-making process of SIBCD. In the present study the evaluation was performed retrospectively and real-life measurements with a measuring tape on the head of the patient were not possible. The head circumference was measured at the CT scan following the method of Vorperian et al. Their group showed that the head diameter can be estimated indirectly from a CT scan with high accuracy when direct measurements with a measuring tape cannot be performed. By applying a non-linear correction factor, the accuracy of the estimation by accounting for nonlinear growth rates and sex differences in head growth can be improved [18].

Another novel aspect of this study was the evaluation for the traditional mastoid implantation site together with two alternative sites, retrosigmoidal and parietal. Nevertheless, when implantation deemed unsuccessful in the traditional site, it was also not possible at the two alternative sites either. To date, only one comparable study exists in the literature which investigated the feasibility of implantation at the mastoidal site in respect to age with the BCI-602 without considering alternative localizations as in the present study [19]. The colleagues investigated a smaller cohort (81 patients), but as in the present study with an also rather evenly distributed share of gender. In addition, the present study compared data of the new BCI-602 and the old BCI-601 in terms of anatomical dimensions and virtual implantation in children and adolescents within the three localizations and, moreover, applied additional predicting parameters, for instance, head diameter. As expected, we observed a more versatile applicability with virtual implantation even in the very young patients under 5 years (to date beyond the range of approval of the SIBCD) with the new device. Regarding the mastoidal localization, this was also reported by Wenzel et al. In addition, they found a safe implantation beyond the age of 12 years in 100% of the cases [19]. The present study could not verify these results. The cutoff threshold was found at an age of 16.5 years for the mastoidal implantation site. Moreover, our study showed the largest difference between the both implants in the age-group of 9–11 years with the new BCI-602 reaching a 78.9% rate of successful implantation and with usage of the 1-mm spacer even a 94.7%-rate. In a virtual analysis with the old BCI-601 Rahne et al*.* found a 100%-rate of applicability with 4-mm lifts in patients older than 8 years [15]. After geometric changes of the device, the same study group compared the virtual implantation of the new BCI-602 to the old device and demonstrated a significantly higher rates of successful implantation with the BCI-602 especially in the age-group of the 3–5 years [19]. In the present study, this age-group was underrepresented with only 8.1% of the whole cohort, which was due to the inadequate resolution and thus large exclusion rate of the screened CT scans in this age-group.

It is well-documented that dimensions of the head and temporal bone grow until the age of 16 [20]. The remaining bone layer to the sigmoid sinus at the mastoidal localization was comparable to results of other studies investigating the bone thickness in children [15, 19, 21]. Comparing the results of the present study to investigations of skull measurements in the retrosigmoid area on specimens, the values were comparable, as well [22]. Regarding the parietal implantation site the present cohort of 0–5 years (*n* = 21) had a slightly thinner skull than the cohort (*n* = 200) of radioanatomy investigation in children for the implantation of a bone anchored hearing device [23].

In particular in very young children, the skin flap covering the implant is often very thin. Regarding the age groups we see at all localizations increasing thickness of skin flap with advancing age. The manufacturer recommends to thin out the skin flap when exceeding a thickness of 7.0 mm to ensure adequate hold of the processor via magnet. Moreover, neuralgia and other pressure-related skin complications can be avoided [24–26]. Nevertheless, thinning out the flap can be accompanied with postoperative complications, such as impaired wound healing and hairless regions. Data regarding those postoperative complications are lacking, to the best of our knowledge. As shown in Fig. 1, the new version of the implant is protruding more than the old one; however, the edges are more smoothed down compared to the old implant. This might be a disadvantage with the new implant in the very small patients with a thinner skin flap. On the other hand, in our cohort this age-group with the thinnest skin flap would require 4-mm spacer with the old BCI-601 to avoid compression of delicate structures, thus leading to protrusion of the implant under the intact skin flap with also more sharper edges than the new version.

Limitations of the study lie in the retrospective nature. The exclusion rate of the screened cohort is quite high with 81.8%, predominately due to the quality deficits of the screened CT scans. Since children react more sensitive to radiation, investigators often try to lower the radiation dosages resulting in a larger slice thickness and inadequate scan-quality [13, 14]. In particular in younger children, the quality is often insufficient, as seen in our cohort with children younger than 5 years representing 15.4% of the cohort. This underlines the importance of the results of the present study that the evaluated parameters (head diameter, gender, age) could aid in decision-making within an evaluation process even prior to CT scan and/or surgery. Another aspect is the limited transferability of this virtual assessment onto a real-life surgical situation. From clinical experience a certain level of compression of the dura mater or sigmoid sinus might be tolerable. In previous published reports the exposure of the dura mater or sigmoid sinus was documented without any following postoperative complications [27–31] Experiences regarding long-term results, however, remain to be evaluated. Within the evaluated cohort all patients with malformations of the temporal bone were excluded from the study. This generated a more homogeneous cohort, however, in a clinical setting patients requiring a bone conduction device exhibit malformations of the temporal bone to a considerable share. Especially for those patients, alternative sites like the retrosigmoidal and parietal localization are adequate options [8–10]. However, the prediction models used in the present study were estimated using moderate sample sizes and larger sample sizes are expected to considerably improve. We further acknowledge that these models are unlikely to be applicable for cases with malformations.

## Conclusion

Geometric changes of the actuator lead to a higher rate of successful implantation in all age groups with reducing the requirement for spacer application. Parameter age and head diameter might help to estimate the successful implantation in the youngest patients and may be a novel tool to assist in the decision-making process for a SIBCD. However, thorough preoperative planning for an SIBCD including imaging remains essential in children and adolescents.

## Supplementary Information

Below is the link to the electronic supplementary material.

Supplemental Figure 1: Head diameter versus age regarding retrosigmoidal implantation with or without spacer with regard to the new BCI-602 or old BCI-601. (A) BCI-601 with 4 mm spacer, (B) BCI-602 with 1 mm spacer, and (C) BCI-602 without spacer. The yellow filled rhombus BCI-601 with spacer (4 mm), blue filled rhombus BCI-602 with spacer (1 mm), the blue empty circles BCI-602 without spacer, and the red cross non-implantable candidates. The horizontal red solid line indicates the cutoff for the head diameter: all values above this line would suggest safe implantation at this site. The vertical red solid line indicates the cutoff for the age: all values beyond this line would suggest safe implantation at this site. No cutoff values for neither head diameter nor age could be identified for a safe implantation. (PDF 43 KB)

Supplemental Figure 2: Head diameter versus age regarding parietal implantation with or without spacer with regard to the new BCI-602 or old BCI-601. (A) BCI-601 with 4 mm spacer, (B) BCI-602 with 1 mm spacer, and (C) BCI-602 without spacer. The yellow filled rhombus BCI-601 with spacer (4 mm), blue filled rhombus BCI-602 with spacer (1 mm), the blue empty circles BCI-602 without spacer, and the red cross non-implantable candidates. The horizontal red solid line indicates the cutoff for the head diameter: all values above this line would suggest safe implantation at this site. The vertical red solid line indicates the cutoff for the age: all values beyond this line would suggest safe implantation at this site. Only for BCI-602 with 1 mm spacer a cutoff value ”age” with 20.2 years could be calculated. For all others no cutoff values for neither head diameter nor age could be identified for a safe implantation. (PDF 44 KB)

Supplementary file3 (PDF 15 KB)

## Data Availability

Original data are available on demand.

## Code availability

For each implantation site, a logistic regression was fitted to model implantation success by age, head size, gender and device (consisting of 3 dummy variables for BCI-602 without spacer, BCI-601 with spacer, and BCI-601 without spacer). The logistic regression was fitted using an iterative reweighted least squares approach using the stat package (version 4.0.5) in the statistical software R (version 4.0.5). Covariates were tested for equality to zero using likelihood ratio test and resulting *p* values were corrected using a Benjamini–Hochberg procedure. For each implantation site, utilizing the caret package (version 6.0-88), we then used the significant covariates to generate a prediction model using a fivefold cross validation to estimate accuracy, sensitivity, and specificity. Note that we only generated predictive models for new technology and separately for the use of spacers and no use of spacers. All figures were created with Microsoft Excel version 1906.
