# Supplementary material for: Parameters for estimating the feasibility of implantation of a semi-implantable bone conduction device (SIBCD) in children and adolescents
Source: Eur Arch Otorhinolaryngol. 2022 Dec 1;280(6):2695–705. doi: 10.1007/s00405-022-07752-6 (PMC10175334; doi:10.1007/s00405-022-07752-6)
Supplement: Supplementary file 1 — Supplemental Figure 1: Head diameter versus age regarding retrosigmoidal implantation with or without spacer with regard to the new BCI-602 or old BCI-601. (A) BCI-601 with 4 mm spacer, (B) BCI-602 with 1 mm spacer, and (C) BCI-602 without spacer. The yellow filled rhombus BCI-601 with spacer (4 mm), blue filled rhombus BCI-602 with spacer (1 mm), the blue empty circles BCI-602 without spacer, and the red cross non-implantable candidates. The horizontal red solid line indicates the cutoff for the head diameter: all values above this line would suggest safe implantation at this site. The vertical red solid line indicates the cutoff for the age: all values beyond this line would suggest safe implantation at this site. No cutoff values for neither head diameter nor age could be identified for a safe implantation. (PDF 43 KB) [file 405_2022_7752_MOESM1_ESM.pdf]

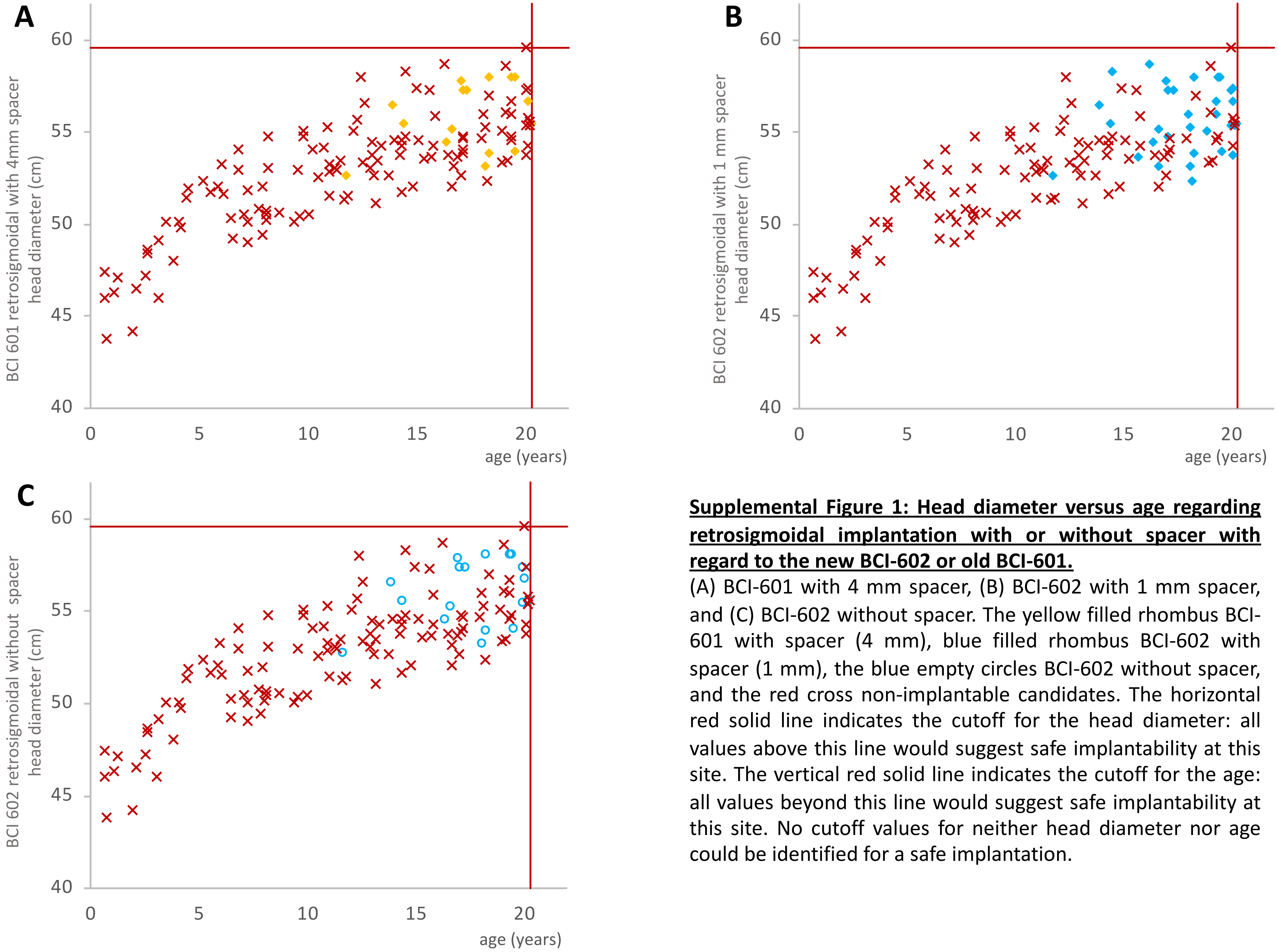

Supplemental Figure 1: Head diameter versus age regarding retrosigmoidal implantation with or without spacer with regard to the new BCI-602 or old BCI-601.

(A) BCI-601 with 4 mm spacer, (B) BCI-602 with 1 mm spacer, and (C) BCI-602 without spacer. The yellow filled rhombus BCI-601 with spacer (4 mm), blue filled rhombus BCI-602 with spacer (1 mm), the blue empty circles BCI-602 without spacer, and the red cross non-implantable candidates. The horizontal red solid line indicates the cutoff for the head diameter: all values above this line would suggest safe implantability at this site. The vertical red solid line indicates the cutoff for the age: all values beyond this line would suggest safe implantability at this site. No cutoff values for neither head diameter nor age could be identified for a safe implantation.
