# Supplementary material for: Parameters for estimating the feasibility of implantation of a semi-implantable bone conduction device (SIBCD) in children and adolescents
Source: Eur Arch Otorhinolaryngol. 2022 Dec 1;280(6):2695–705. doi: 10.1007/s00405-022-07752-6 (PMC10175334; doi:10.1007/s00405-022-07752-6)
Supplement: Supplementary file 3 — Supplementary file3 (PDF 15 KB) [file 405_2022_7752_MOESM3_ESM.pdf]

Supplemental Material

Supplemental Table: Exclusion Criteria

Exclusion Criteria		
	Number	(%)
Clinical diagnosis	195	(32.0)
Inner or middle ear malformation		
Visual assessment	61	(10.0)
Preexisting implants or other artefacts, dislocated fracture within the temporal bone or adjacent skull base, abnormal pneumatization		
Slice thickness > 0.6 mm	152	(24.9)
Multiple CT scans per patient (one patient included once only)	202	(33.1)
Total number of screened data sets	746	(100.0)
Included cases	136	(18.2)

CT, computer tomography.
